# Long-term maintenance of human induced pluripotent stem cells by automated cell culture system

**DOI:** 10.1038/srep16647

**Published:** 2015-11-17

**Authors:** Shuhei Konagaya, Takeshi Ando, Toshiaki Yamauchi, Hirofumi Suemori, Hiroo Iwata

**Affiliations:** 1 Department of Regenerative Materials, Institute for Frontier Medical Sciences, Kyoto University, 53 Kawahara-cho, Shogoin, Sakyo-ku, Kyoto 606-8507, Japan; 2 Global Manufacturing Division, Panasonic Corporation, 2-7 Matsuba-cho, Kadoma City, Osaka 571-8502, Japan; 3 Department of Embryonic Stem Cell Research, Institute for Frontier Medical Sciences, Kyoto University, 53 Kawahara-cho, Shogoin, Sakyo-ku, Kyoto 606-8507, Japan

## Abstract

Pluripotent stem cells, such as embryonic stem cells and induced pluripotent stem (iPS) cells, are regarded as new sources for cell replacement therapy. These cells can unlimitedly expand under undifferentiated conditions and be differentiated into multiple cell types. Automated culture systems enable the large-scale production of cells. In addition to reducing the time and effort of researchers, an automated culture system improves the reproducibility of cell cultures. In the present study, we newly designed a fully automated cell culture system for human iPS maintenance. Using an automated culture system, hiPS cells maintained their undifferentiated state for 60 days. Automatically prepared hiPS cells had a potency of differentiation into three germ layer cells including dopaminergic neurons and pancreatic cells.

Such pluripotent stem cells (PSCs) as embryonic stem (ES) cells and induced pluripotent stem (iPS) cells undergo self-renewal and differentiate into multiple types of functional cells^1^,^2^. Due to their properties, these cells are regarded as alternative sources for pharmaceutical researches and cell replacement therapy^3^,^4^,^5^,^6^. Recently, phase I/II clinical trials have begun on treatments for type I diabetes^7^ and age-related macular degeneration^8^,^9^ using ES cells. The use of PSCs will continue to increase in the future.

Automated culture systems enable the large-scale production of cells^10^,^11^. In addition to reducing the time and effort of researchers, automated culture systems improve the reproducibility of cell cultures. Compared with other types of cells, such as cancer cells, the maintenance of hiPS cells is considered technically difficult due to the instability of their undifferentiated state and the sensitivity of mechanical stress^1^,^2^,^12^,^13^. In other words, the quality of hiPS cells strongly depends on technician skill. Although automated cell culture systems have been reported for the maintenance of PSCs, the characterization of automatically cultured PSCs remains inadequate^14^,^15^,^16^,^17^. This is because it is difficult to keep long-term stability of the undifferentiated state of PSCs from the viewpoint of technical level of robotics and automation.

In the present study, we newly designed a fully automated cell culture system that automates cell seeding, medium changing, cell imaging, and cell harvesting. The automated cell culture system’s movement, which was designed based on the video analysis of an expert’s culture operation, focused on the passage procedure. Using our automated system, we cultured human iPS cells on feeder cells for sixty days and twenty passages. We evaluated the pluripotency of the automatically expanded hiPS cells, especially the expression of the pluripotent markers and the capability of differentiation into specific types of cells including dopaminergic neurons and pancreatic islet cells.

## Results

### Design of automated cell culture system for human iPS cell maintenance

**Figure 1:**
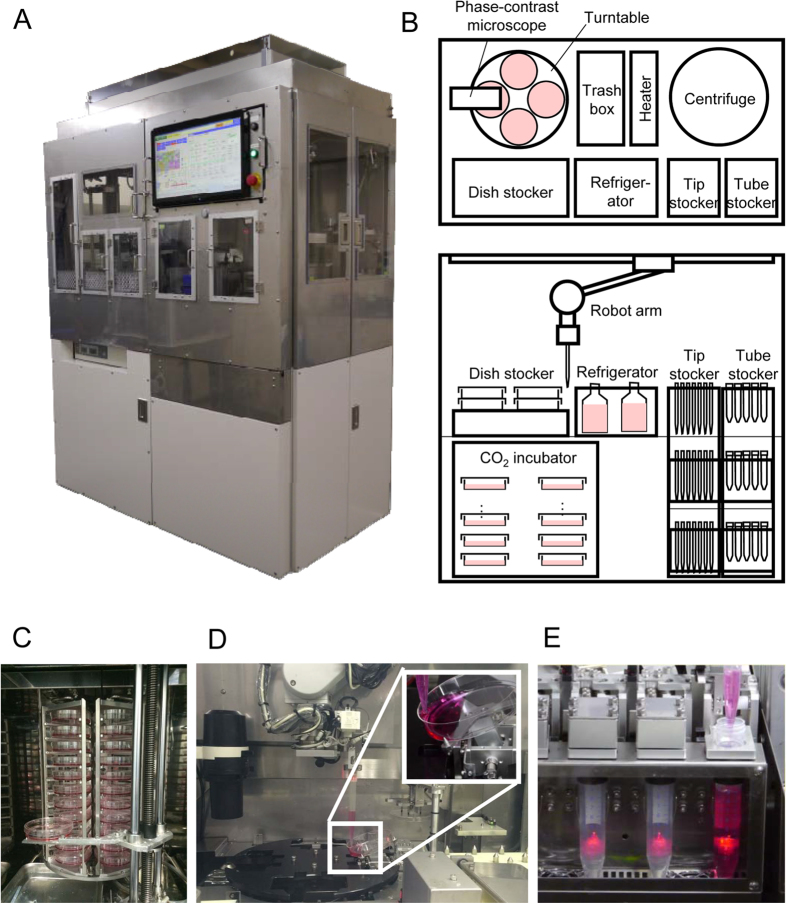
Automated culture system of hiPS cells. (**A**) Outside view of automated culture system. (**B**) Module layout of automated culture system. (**C–E**) Photographs of cell culture modules. (**C**) CO_2_ incubator, (**D**) Turntable and robotic arm, (**E**) Heater.

**Figure 2:**
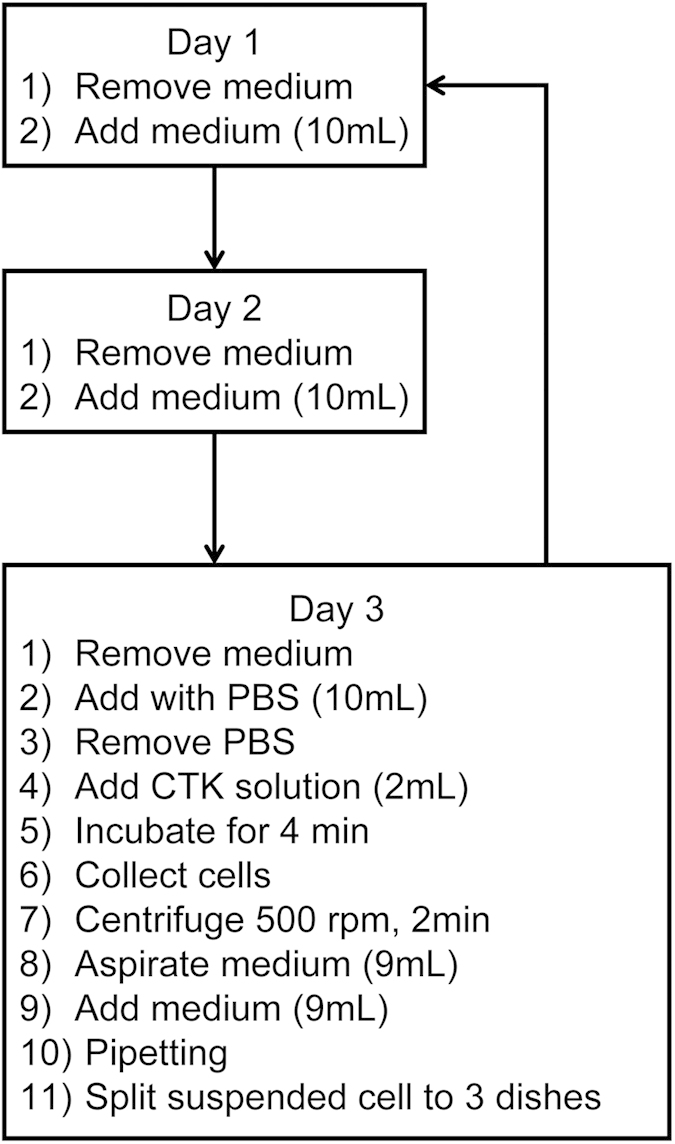
Procedure diagram of automated culture of hiPS cells. Days 1–2: Changing cell culture media. Day 3: Passaging. CTK: 0.25% (v/v) trypsin and 0.1 mg/mL collagenase in PBS (-) supplemented with 20% (v/v) KSR and 1-mM calcium chloride.

Based on the timing which is pre-set using a PC’s touch panel display, medium changes and passage procedures were automatically conducted using our automated culture system (Fig. 1A and Supplementary movie 1). As shown in Fig. 2, culture medium was changed every day and hiPS cells were subcultured every three days. The passaging procedures of one culture dish takes approximately 40 minutes. The passage procedures were totally conducted twenty times. During this long-term experiment, the sanitation level inside the automated culture system maintained a clean class of 100, which is defined as the number of particles whose size exceeded 0.1 μm was less than 100/m^3^.

Users controlled the automated cell culture system using the touch panel of a PC. They set the timing of the medium changes and the passages of every dish using the scheduler displayed on the touch panel, which also shows such system conditions as alarms, culture schedules, and environmental conditions, including the CO_2_ incubator, refrigerator, and heater temperatures, and the residual quantities of media, the remover, the pipet tips, and the centrifuge tubes. Users can confirm the culture conditions in real-time.

The cells in the culture dishes are kept and incubated in the CO_2_ incubator except for the timing of medium change and passage procedure (Fig. 1D). At the timing of the medium changes or passages, the cultured dishes are automatically transferred and set on a turntable. The robot arm to which a pipet tip was attached adds or aspirates the reagent of the dishes set on the turntable. Depending on the task of the cell culture, the robot arm automatically changes its tip attachments (dish-handling tool, pipet tool, or centrifuge-tube handling tool) to complete many different tasks of medium changes and passages. The dish-handling tool transfers the dishes from the incubator to the turntable or vice versa. The pipet tool attaches or removes the pipet tip to transfer the reagent from the bottle in the refrigerator to the centrifuge tube in the heater from the centrifuge tube in the heater to the dish on the turntable and aspirates the reagent from the dish. The tube-handling tool opens the cap of the tube, sets the tube in the heater, and transfers it from the heater to the centrifuge.

After the medium change and passage procedures, the images of the phase-contrast microscope are automatically recorded to confirm cell growth and quality. The recorded images and following environmental data are stored in the PC: incubator CO_2_/temperature conditions, heater and refrigerator temperatures, air flow velocity, and time stamps of each task.

Supplementary figure 1 shows a schematic illustration of the splitting procedure. HiPS cells in fifteen dishes were split into forty-five MMC-treated feeder dishes. The MMC-treated feeder dishes were set in an incubator before the splitting. After 3 days of culture, thirty of forty-five dishes with hiPS cells were supplied to the users. HiPS cells in the remaining fifteen dishes were used for the splitting procedure. Therefore, our automated cell culture system supplied thirty dishes with hiPS cells every three days. The used consumables and non-used hiPS dishes were discarded in a trash bag.

From video analysis of an expert’s manual operations, we identified the following critical factors that affect the quality of hiPS cells: 1) position and direction of the pipet tip for pouring fresh medium during the medium change; and 2) pipetting velocity to break the hiPS colonies in the passage procedure. In the former, fresh medium was poured along the sidewall of the dish on the turntable (Fig. 1E). In the latter, the pipetting velocity was set to 5.0 mL/s to imitate the expert’s techniques (Fig. 1F).

### Automatically cultured hiPS cells expanded as manually cultured hiPS cells

**Figure 3:**
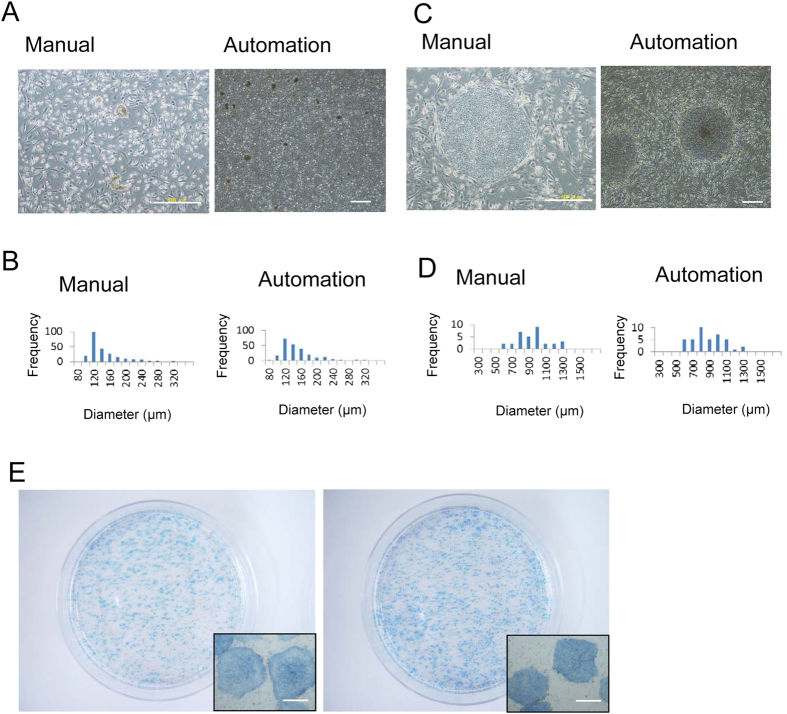
Automated passaging of hiPS cells. (**A,C**) Phase micrographs of hiPS cells immediately after seeding (**A**) and after three days (**C**). (**B,D**) Distribution of hiPS colony size immediately after seeding (**B**) and after three days (**D**). HiPS colony size were analyzed from their phase contrast images. Y axis indicates the number of the colonies. At least 50 images were used for the measurement. (**E**) Alkaline phosphatase staining of hiPS cells on day 3. Scale bar: 500 μm. urpp.

One of the technical difficulties of managing hiPS cultures is the passaging procedures. Generally, hiPS clumps (hiPS colonies) must be divided into appropriate sized small clumps before seeding on the culture surface^1^,^2^. Excessively small colonies and fully dissociated hiPS cells tend to die by apoptosis due to their sensitivity to mechanical stress and single cell dissociation^12^,^13^. As manual passaging procedures, hiPS cells were automatically divided by pipetting using plastic tips (Fig. S1). The shape of the tips used for the culture system was almost the same as the conventional plastic tips except for an elongated nozzle that prevents liquid from spilling. With no additional specific device for passaging, the passaging methods simplify the configuration of the automated culture system and provide high availability for multiple culture methods. CTK solution, an enzymatic cocktail composed of collagenase, trypsin, and knockout serum replacement, was used for the passaging. HiPS 253G1 line was mainly used in this study to assess the feasibility of the automated culture system. Figure 3A contains phase-contrast images of manually and automatically divided clumps of hiPS cells. The size of divided clumps was controlled by the pipetting velocity (Fig. S2). By adjusting the pipetting number and speed, the hiPS cell clumps are automatically divided into small clumps whose size resembles manually divided hiPS clumps. The average size of the manually and automatically divided hiPS clumps was 136 ± 39.9 μm and 139 ± 40.8 μm, respectively (Fig. 3B).

Next, hiPS cells were automatically cultured for three days on feeder cells. The automatically cultured hiPS cells proliferated like manually cultured hiPS cells and exhibited typical morphology (Fig. 3C). After being cultured for three days, the average colony size of the manually and automatically cultured hiPS increased to 966 ± 211 μm and 919 ± 198 μm, respectively (Fig. 3D). This indicates that the growth rate of the automatically cultured hiPS was equivalent to the manually cultured hiPS cells. In both cultures, the hiPS colonies were positive for alkaline phosphatase staining (Fig. 3E). Note that the alkaline phosphatase positive-hiPS colonies were homogeneously distributed on the culture surface. Heterogeneous cell adhesion was avoided by minimalizing the mechanical vibration from the culture system. For example, we arranged an incubator separate from a centrifugal machine. Dispase, as a cell detachment reagent, was also examined for the passaging procedures. HiPS cell clumps were automatically divided into appropriate sized small clumps using Dispase. The cells proliferated on feeder cell and expressed pluripotent cell markers (Fig. S3).

### HiPS cells maintained their undifferentiated state by automated culture systems for a long period

**Figure 4:**
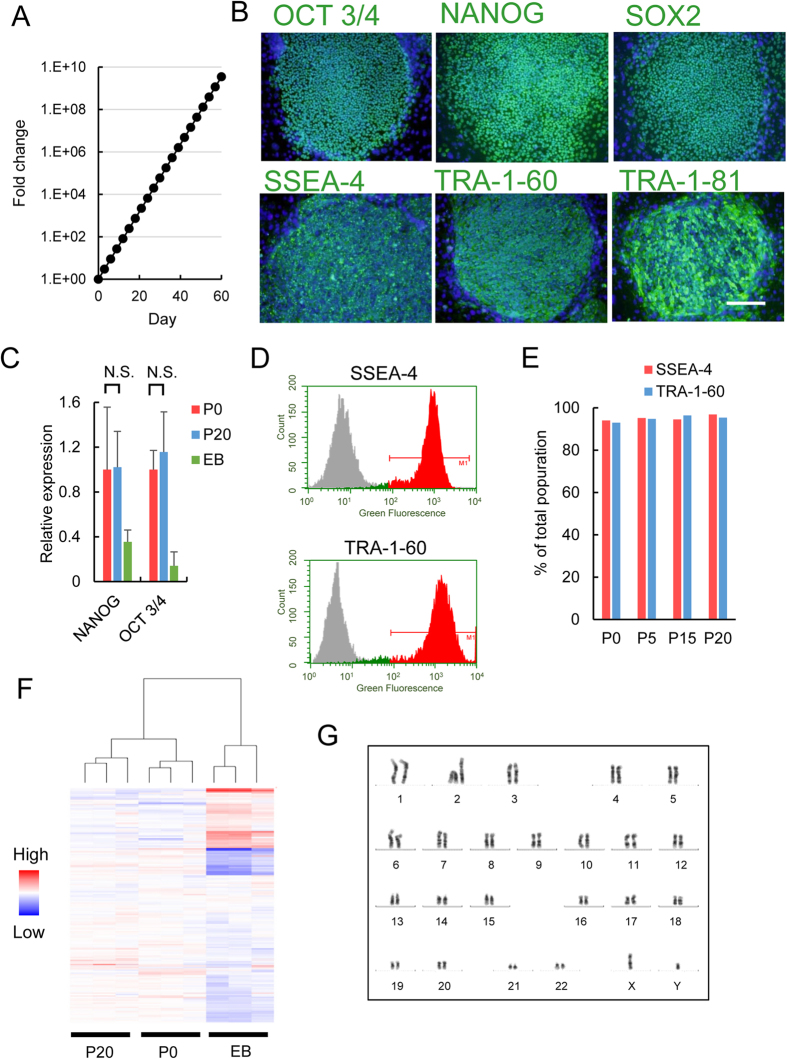
Long-term culture hiPS cells by automated system. (**A**) Fold change of cell number determined by split ratio. Black circles indicate each passaging point. (**B**) Fluorescent micrograph of hiPS cells automatically passaged 20 times. Cells were immunologically stained with antibodies against OCT 3/4, NANOG, SOX2, SSEA-4, TRA-1-60, and TRA-1-81. Cell nuclei were stained with Hoechst 33258. Scale bar: 200 μm. (**C**) qPCR analysis of gene expression of OCT 3/4 and NANOG. Expression levels were normalized to expression levels of unpassaged cells (mean ± sd., n = 3). P0, unpassaged cells; P20, cells automatically passaged for 20 times; EB, embryoid body. N.S., not significant. (**D**) Representative histograms of FACS analysis. (**E**) FACS quantification of cells throughout culture. (**F**) Global gene expression analysis. Cluster analysis was performed with all detected genes (**G**) Karyotype analysis of hiPS cells automatically passaged 20 times.

HiPS cells (253G1 line) were automatically cultured for 60 days to evaluate their long-term stability. Every three days, the hiPS cells were automatically passaged on SNL feeder cells. During the culture period, they were expanded without decreasing their growth rate. The number of cells virtually increased 10^10^-fold by the end of the culture period (Fig. 4A). Figure 4B shows immunofluorescent images of the hiPS cells automatically cultured for 60 days. The cells expressed transcript factors, such as OCT 3/4, NANOG, and SOX2. The expression levels of OCT 3/4 and NANOG did not change after 20 passages (Fig. 4C). Additionally, the cells were positive for cell surface markers, TRA-1-60, TRA-1-81, and SSEA-4. FACS analysis revealed that approximately 95% were positive for TRA-1-60 and SSEA-4 throughout the culture period (Fig. 4D,E). Global gene expression analysis (SurePrint G3 Human Gene Expression 8 × 60 K v2, Agilent Technologies, Santa Clara, CA, USA) was performed with a microarray. The hiPS that were automatically passaged 20 times (P20 hiPS cells) exhibited similar expression patterns as the unpassaged hiPS cells (P0 hiPS cells) (Fig. 4F and Fig. S4). We found a very close concordance of the averaged-global gene expression between the P0 and P20 hiPS cells (R^2^ = 0.99, Fig. S4). The hiPS cells had normal karyotypes after long-term culture (Fig. 4G). HiPS 253G1 line was used in the above mentioned examinations. The other two hiPS cell lines, 454E2 and RIKEN-2A, were also expanded by the automated culture system and maintained their undifferentiated state (Fig. S5).

### Automatically cultured hiPS cells successfully differentiated into three germ layers

**Figure 5:**
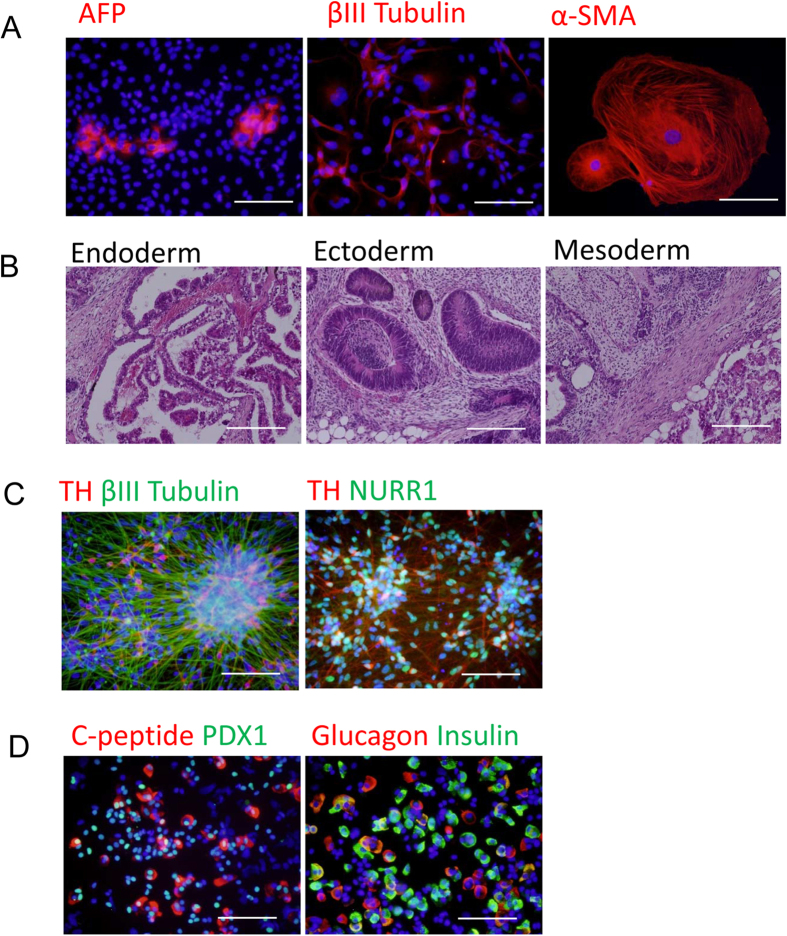
Differentiation of automatically cultured hiPS cells into three germ layers. HiPS cells were automatically passaged for 20 times and subjected to differentiation conditions. (**A**) Embryoid body-mediated differentiation of automatically cultured hiPS cells. Cells were cultured for two weeks in differentiation medium and stained with antibodies against AFP, βIII-Tubulin, and α-SMA. (**B**) Hematoxylin & Eosin staining of teratoma. Teratoma was extracted from immunodeficient mouse 2 mounts after injection of hiPS. (**C**) Directed differentiation of automatically cultured hiPS cells into dopaminergic neurons. Cells were immunologically stained with antibodies against tyrosine hydroxylase (TH), βIII-Tubulin, and nuclear receptor related 1 protein (NURR1). (**D**) Directed differentiation of automatically cultured hiPS cells into pancreatic cells. Cells were immunologically stained with antibodies against PDX1, C-peptide, Insulin, and Glucagon. Scale bar: 100 μm.

After long-term culture, we manually differentiated hiPS cells into three germ layer cells to evaluate their pluripotency. First, the hiPS cells were differentiated through EB formation *in vitro*. As shown in Fig. 5A, they were differentiated into AFP-positive endoderm cells, βIII-Tubulin-positive ectoderm cells, and α-SMA-positive mesoderm cells after two weeks of differentiation. The hiPS cells formed teratoma and differentiated into three germ layer cells two months after being injected into an immunodeficient mouse (Fig. 5B).

Then we differentiated the hiPS cells into specific types of cells. In our experiments, we differentiated P20 hiPS cells into dopaminergic neuron and pancreatic islet cells. The cells were differentiated into TH- and NURR1-positive dopaminergic neurons after 25 days of differentiation (Fig. 5C). They were also differentiated into PDX1-positive pancreatic cells and a considerable number were positive for pancreatic hormones, such as Insulin and Glucagon. The efficiency of differentiation was roughly the same as in our previous studies^18^.

## Discussions

We developed an automated culture system for the maintenance of hiPS cells, whose undifferentiated state was maintained for 60 days by the system (Fig. 4). After 20 passagings, the hiPS cells had a potency of differentiation into three germ layer cells, including dopaminergic neurons and pancreatic cells (Fig. 5).

Compare with other automated culture system^14^,^15^,^16^,^17^,^19^, our system is more compact in size and has high flexibility for multiple culture methods. In the present study, hiPS cells were cultured on feeder cells in a culture medium containing a knockout serum replacement, which is a gold standard method of hiPS culture^1^,^2^,^20^,^21^. Our automated culture systems are based on the procedures of manual cell culture methods and use conventional cell culture dishes. Each process, such as adding and aspirating liquid, can be freely combined. Although we did not show other culture methods, our automated culture systems can be potentially applied with them. We plan to culture hiPS cells in feeder-free cultures and sequentially differentiate them into pancreatic cells.

One advantage of automated cultures is the traceability of each cell culture. A cell culture dish is placed in an individual position to reduce the risk of cross-contamination. The information of cell culture procedures such as types of culture media is also stored in the system. Researchers can easily track the history of individual cell cultures. Furthermore, hiPS colonies can be tracked by taking images of the specific positions of cell culture surfaces. At most 500 positions (3.2 mm × 3.8 mm) on a culture dish can be tracked under a phase contrast microscope in the culture system. Our culture system is loaded with an automated-detection tool of hiPS colonies that detects the area of the hiPS colonies on the culture surface and predicts the growth rate of the cells to a certain degree. Based on their morphological feature, hiPS colonies were distinguished from feeder cells. In the future, we will improve the analytical tool and design an automated judgment system of the undifferentiated state of hiPS cells.

## Methods

### Design of an automated culture system

As shown in Fig. 1B, our automated culture system is composed of a 6-axis robot arm (MELFA, RV-4FC-D, Mitsubishi, Tokyo, Japan), a CO2 incubator (IT400, Yamato, Japan, capacity: sixty 10-cm dishes), a refrigerator (capacity: six 500-ml bottles and eight 15-ml tubes, temperature: 4 °C), a heater (capacity: four 15-ml tubes, temperature: 37 °C), a centrifuge (rotational velocity: 0 ~ 2000 rpm), a phase-contrast microscope (4X, Nikon, Tokyo, Japan), a trash box, storage areas for dishes (Falcon, Corning, New York, USA, diameter: about 10 and 60 mm), pipet tips (capacity: 225 tips) and centrifuge tubes (capacity: 200 tubes). The system is 1550 mm wide, 1100 mm deep, and 2250 mm high. The motion and condition of all devices and units are controlled by the PC embedded in the automated culture system.

The following are the arrangement features of the automated culture system: 1) such consumables as dishes, bottles, pipet tips, and centrifuge tubes are easily supplied from its front side, 2) a turntable to add/aspirate reagent, a refrigerator, a heater, and a trash box are located close together to decrease the liquid handling distance to prevent spilling the liquid from the pipet tip, and 3) the incubator is separated from the incubator so that the vibration of the centrifuge is not conveyed.

Such consumables as the medium, removers, pipet tips, and centrifuge tubes are manually set every five days. The MMC-treated feeder dishes are supplied manually to the automated cell culture system.

### HiPS cell culture

253G1 line (passage number: P32 – P38), 454E2 line (P34), and RIKEN-2A line (P19) (RIKEN Cell Bank, Ibaraki, Japan.) were used in this study. All experiments were performed in accordance with ethical guidelines of our university and the cell bank. Undifferentiated hiPS were maintained on SNL 76/7 cells (ECACC, Salisbury, UK) as a feeder layer, as previously described^18^. The undifferentiated hiPS cells were cultured in Dulbecco’s modified Eagle medium/F12 (DMEM/F12, Sigma-Aldrich, St. Louis, MO, USA) supplemented with 20% (v/v) knockout serum replacement (KSR, Life Technologies, Carlsbad, CA, USA), 0.1 mM nonessential amino acid (NEAA, Life Technologies), 2 mM L-glutamine (Sigma-Aldrich), 0.1 mM 2-mercaptoethanol (Nacalai Tesque, Kyoto, Japan), and 5 ng/mL basic fibroblast growth factor (bFGF, Wako Pure Chemical Industries, Osaka, Japan) in a humidified atmosphere of 5% CO_2_ and 95% air at 37 °C. The hiPS cells were subcultured every three days using CTK solution (0.25% (v/v) trypsin and 0.1 mg/mL collagenase (Nitta Gelatin Inc., Osaka, Japan) in PBS (-) supplemented with 20% (v/v) KSR and 1-mM calcium chloride). The details of the automated hiPS culture are described in Fig. 2. The manual culture was conducted by an expert with five years of experience with hiPS culture.

### Immunocytochemistry

Antibodies used for the immunohistochemistry are listed in Supplementary Table 1. The cells were fixed with 4% paraformaldehyde in PBS for 20 min at room temperature and treated with a 0.2% Triton X-100 solution for 15 min at room temperature to permeabilize the cells. Then they were treated with Blocking One reagent (Nacarai Tesque) for 90 min to block the non-specific adsorption of the antibodies. The antibody solutions were applied to the cells and incubated for 2 h at room temperature. After washing with PBS containing 0.05% Tween 20, the cells were treated with fluorescent-labeled secondary antibodies at a dilution of 1:500 for 1 h at room temperature and washed with PBS containing 0.05% Tween 20. The cell nuclei were counterstained with 1 μg/mL Hoechst 33258 (Dojindo Laboratories, Kumamoto, Japan). The localization of the secondary antibodies was analyzed with a fluorescent microscope (BX51 TRF, Olympus Optical Co., Ltd., Tokyo, Japan).

### Flow cytometry

HiPS cells were dissociated into single cells by treatment with TrypLE select (Life Technologies) and blocked with 1% BSA to prevent non-specific adsorption of the antibody. The cells were reacted with antibodies against SSEA-4 (1:200, mouse monoclonal; Millipore) or TRA-1-60 (1:200, mouse monoclonal; Cell Signaling Technologies) for 30 min at 4 °C and washed with phosphate-buffered saline containing 1% BSA to remove the unreacted antibody. Subsequently, the cells were reacted with a secondary antibody (Alexa Fluor 488 anti-mouse IgG or Alexa Fluor 488 anti-mouse IgM antibody (Life Technologies)) for 30 min at 4 °C and washed with phosphate-buffered saline containing 1% BSA to remove the unreacted antibody. The population of the fluorescently active cells was analyzed using a Guava EasyCyte Mini flow cytometer (Millipore) equipped with a 488-nm diode laser. We used the data from approximately 10,000 cells to generate a histogram. Cells harvested from the substrates and exposed only to the secondary antibody were used as negative controls. Data from the control experiments were used to set the threshold for identifying the SSEA-4- or TRA-1-60-expressing cells.

### Quantitative PCR

After 20 passagings, the hiPS cells were collected using a CTK solution. The total RNA was extracted with the SV Total RNA Isolation System (Promega Corp., Madison, WI, USA). The first strand cDNA was prepared from the RNA by reverse transcription using the Transcriptor First Strand cDNA Synthesis Kit (Roche Applied Science, Mannheim, Germany) and oligo(dT)18 primers as a RT primer. Real-time PCR reaction was carried out using StepOne™ Real-Time PCR Systems (Life Technologies). The reaction mixtures (20 μL), which contained a Power SYBR Green PCR Master Mix (Life Technologies) of a 200 ng cDNA template, a 50 nM sense primer, and a 50 nM antisense primer, were subjected to PCR. The primers used for amplification are listed in Supplementary Table 2. The expression levels of each gene were normalized to the GAPDH expression. As a reference, we used EB-mediated differentiated cells for this study and averaged the results of three samples. The data, which are shown as mean ± standard deviation (sd) for three independent samples, were compared by Student’s t-test. All the statistical calculations were performed using JMP software (SAS Institute Inc., NC).

### Embryoid body (EB) -mediated differentiation

The hiPS cells were harvested from culture dishes by a CTK solution. The cells were cultured in DMEM (Sigma-Aldrich) supplemented by 10% FBS for one week to form EBs, which were allowed to adhere on the Gelterx^TM^ (Life Technologies)-coated surface. After a one-week culture, the cells were fixed with PFA and immunofluorescently stained.

### Teratoma formation *in vivo*

All animal experiments were carried out according to the guidelines of our institute’s Animal Welfare Committee. All experimental protocol were approved by the committee. Automatically cultured hiPS cells (P20) were collected from the cultured substrates and injected into immunodeficient mice.

## Additional Information

**How to cite this article**: Konagaya, S. *et al*. Long-term maintenance of human induced pluripotent stem cells by automated cell culture system. *Sci. Rep*. **5**, 16647; doi: 10.1038/srep16647 (2015).

## Supplementary Material

Supplementary Information

Supplementary Movie S1
