# Supplementary material for: Long-term maintenance of human induced pluripotent stem cells by automated cell culture system
Source: Sci Rep. 2015 Nov 17;5:16647. doi: 10.1038/srep16647 (PMC4647834; doi:10.1038/srep16647)
Supplement: Supplementary Information [file srep16647-s1.pdf]

Supplementary Information

Long-term maintenance of human induced pluripotent stem cells by automated cell culture system

Shuhei Konagaya[1], Takeshi Ando[2], Toshiaki Yamauchi[2], Hirofumi Suemori[3], and Hiroo Iwata[1]*

[1]Department of Regenerative Materials, Institute for Frontier Medical Sciences, Kyoto University, 53 Kawahara-cho, Shogoin, Sakyo-ku, Kyoto 606-8507, Japan
[2]Global Manufacturing Division, Panasonic Corporation, 2-7 Matsuba-cho, Kadoma City, Osaka 571-8502, Japan
[3]Department of Embryonic Stem Cell Research, Institute for Frontier Medical Sciences, Kyoto University, 53 Kawahara-cho, Shogoin, Sakyo-ku, Kyoto 606-8507, Japan

*Correspondence should be addressed to Hiroo Iwata
E-mail: iwata@frontier.kuoyo-u.ac.jp
Phone: +81-75-751-4119
Fax: +81-75-751-4646

Materials and Methods

Alkaline phosphatase staining

Automated and manually cultured hiPS cells were fixed with 4% PFA in PBS for 2 min at room temperature. The cells were stained with VECTOR Blue Alkaline Phosphatase Substrate Kit (Vector Laboratories, Inc., Burlingame, CA, USA) in accordance with the manufacturer's instructions.

Differentiation of hiPS cells into dopaminergic neurons

Undifferentiated hiPS cells were seeded on Matrigel (BD Bioscience, San Jose, CA, USA) coated cell culture dishes. Cells were cultured in a DMEM/F12 medium (Life Technologies) supplemented with 2.5 mM GlutaMax, 15% KSR, and 0.1 mM 2-mercaptoethanol (Nacalai Tesque) at 37°C under 5% CO_2. Ten micromolar SB 431542 (Wako), 100 nM LDN193189 (Wako), 3 μM CHIR99021 (Wako), 100 ng/mL sonic hedgehog N-terminus (R&D Systems), 2 mM purmorphamine (Wako), and 100 ng/mL fibroblast growth factor-8 (Wako) were added to a culture medium in a time-dependent fashion. KSR was gradually shifted to N2 supplement (Life Technologies) from days 5 to 11. On day 11, the culture medium was changed to DMEM/F12 that was supplemented with a 2.5 mM GlutaMax, a 2% B27 supplement (Life Technologies), a 10 ng/mL brain-derived neurotrophic factor (Wako), a 10 ng/mL glial cell line-derived neurotrophic factor (Wako), a 0.2 mM ascorbic acid (Nacalai Tesque), a 0.5 mM dibutyryl cyclic adenosine monophosphate (Nacalai Tesque), a 1 ng/mL transforming growth factor β3 (R&D Systems), 100 U/mL penicillin, and 100 μg/mL streptomycin (Nacalai Tesque). Cells were cultured for an additional week for the maturation of neuronal cells. On day 18, cells were subcultured on a laminin /poly-L-ornithine-coated dish for further maturation. On day 25, they were fixed with PFA in PBS and immunologically stained.

Differentiation of hiPS cells into pancreatic β cells

HiPS cells were a subcultured Geltex-coated culture surface and cultured in E8 medium (Life Technologies) for 3-4 days. At 60-70% of confluence, the cells were treated with TrypLE (Life Technologies) for 5 min at room temperature. The released single cells were collected and centrifuged at 1000 rpm for 5 min and resuspended with an E8 medium containing 10 μM Y-27632 (Wako). The cells were seeded into agarose microwell plates (Microtissues Inc., Providence, RI) at a density of 2500 cells/well and cultured for 24 h to induce aggregation. The formed cell aggregates were cultured for 34 days to induce differentiation into pancreatic lineage and allowed to adhere on a Geltrex-coated surface

for immunostaining. The culture mediums were daily changed according to the following time schedule:

Stage 1 (days 1-3): RPMI + 1.2 g/L Sodium bicarbonate + 0.1% fat-free BSA (Wako) + 1/5000 ITS supplement (Life Technologies) + 3 μM CHIR99021 (Wako) + 100 ng/mL Activin A (R&D systems). CHIR99021 was only added to the culture medium on the first day.

Stage 2 (days 4-6): DMEM/F12 + 0.1% fat-free BSA + 1/5000 ITS supplement + 50 ng/mL FGF-7 (PeproTech)

Stage 3 (days 7-10): DMEM + 1% B27 supplement (Life Technologies) + 50 ng/mL FGF-7 + 0.25 μM SANT-1 (Wako) + 0.5 μM LDN 193189 (Wako) + 2 μM retinoic acid (Sigma-Aldrich).

Stage 4 (days 11-13): DMEM + 1% B27 supplement + 0.25 μM SANT-1 + 0.5 μM LDN 193189 + 0.5 μM PdBu (Sigma-Aldrich).

Stage 5 (days 14-20): DMEM + 1% B27 supplement + 1 μM Alk5 inhibitor (Wako) + 0.25 μM LDN 193189.

Stage 6 (days 20-34): DMEM + 1% B27 supplement.

DNA microarray

The total RNA was isolated from the hiPS cells at P0, hiPS at P20, and EBs. Global gene expression analysis was performed with SurePrint G3 Human Gene Expression 8x60K v2 (Agilent Technologies, Santa Clara, CA, USA). The expression levels of each sample were analyzed by GeneSpring13 (Agilent Technologies).

Discussions

During a long-term culture of human ES/iPS cells, some colonies are spontaneously differentiated. The numbers of undifferentiated and differentiated cells are calculated as follows. Initial number of undifferentiated cells, which is applied to a dish at the start of cell culture, is assumed to be A. The cells in a dish were split to three dishes every 3 days in the present study. The number of total cells increases to 3A. The cells contain 3Aa differentiated cells and 3A(1-a) undifferentiated cells, here, a is probability of differentiation of undifferentiated cells during 3 days. In the next 3 days, the numbers of undifferentiated and differentiated cells become $3^2A(1-a)^2$ and $3^2A(1-a)a + 3Aab$, respectively, here, b is the cell number increase rate of differentiated cells during 3 days culture. The numbers of undifferentiated and differentiated cells at different subculture is given by the following equations:

Numbers of undifferentiated cells = $3^n A(1-a)^n$

Numbers of differentiated cells = $3^nAa(1-a)^{n-1} + 3^{n-1}Aab(1-a)^{n-2} + 3^{n-2}Aab^2 (1-a)^{n-3} + \cdots$

$$= Aab^n\sum_{i=1}^{i=n}(3/b)^i(1-a)^{i-1}$$

The ratios of numbers of undifferentiated and total cells are plotted in Supplementary figure 6. a = 0.02 and 0.05 mean 2% and 5% of undifferentiated cells differentiate after 3 days culture, respectively. b' is 3/b, that is, the ratio of growth rates of undifferentiated and differentiated cells. When the growth rate of undifferentiated cells is larger than that of differentiated cells, b' = 3/b > 1, the ratios of undifferentiated and total cells levels off after about 10 times subcultures. On the other hand, the ratios continuously decrease, when the growth rate of undifferentiated cells is smaller than that of differentiated cells, b' = 3/b < 1. These results indicate that undifferentiated cells are major cells even after infinite times subcultures when b' = 3/b > 1, but differentiated cells became major cells when b' = 3/b < 1. In our culture, the ratios of undifferentiated and total cells were kept 0.93 ~ 0.97 during a long-term culture as shown in Figure 4E. These results suggest that probability of differentiation of undifferentiated cells is a = ~ 0.03 and ratio of growth rates of undifferentiated and differentiated cells is b' = 2.

Supporting Table 1. List of antibodies used for immunofluorescence staining

Antigen	Host	Dilution	Manufacturer
OCT 3/4	Rabbit	1:50	Santa Cruz Biotechnology, Inc., Santa Cruz, CA
NANOG	Rabbit	1:100	Cell Signaling Technology, Danvers, MA
SOX2	Rabbit	1:100	Cell Signaling Technology, Danvers, MA
TRA-1-60	Mouse	1:200	Cell Signaling Technology, Danvers, MA
TRA-1-81	Mouse	1:200	Cell Signaling Technology, Danvers, MA
SSEA-4	Mouse	1:200	Merck Millipore, Billerica, MA
AFP	Mouse	1:200	Sigma-Aldrich, St. Louis, MO
β-Tubulin III	Rabbit	1:500	Covance, Princeton, NJ
α-SMA	Mouse	1:200	Sigma-Aldrich, St. Louis, MO
TH	Mouse	1:200	Merck Millipore, Billerica, MA
NURR1	Rabbit	1:100	Santa Cruz Biotechnology, Inc., Santa Cruz, CA
PDX1	Goat	1:200	R&D systems, Minneapolis, MN
C-peptide	Rabbit	1:100	Cell Signaling Technology, Danvers, MA
Insulin	Guinea pig	1:100	Abcam, Cambridge, UK
Glucagon	Mouse	1:200	Sigma-Aldrich, St. Louis, MO

Supporting Table 2. List of primer sequences used for qPCR analysis

Primer name	Sequence
NANOG forward	CAGAAGGCCTCAGCACCTAC
NANOG reverse	ATTGTTCCAGGTCTGGTTGC
OCT 3/4 forward	CAGTGCCCGAAACCCACAC
OCT 3/4 reverse	GGAGACCCAGCAGCCTCAAA
GAPDH forward	GTGGACCTGACCTGCCGTCT
GAPDH reverse	GGAGGAGTGGGTGTCGCTGT

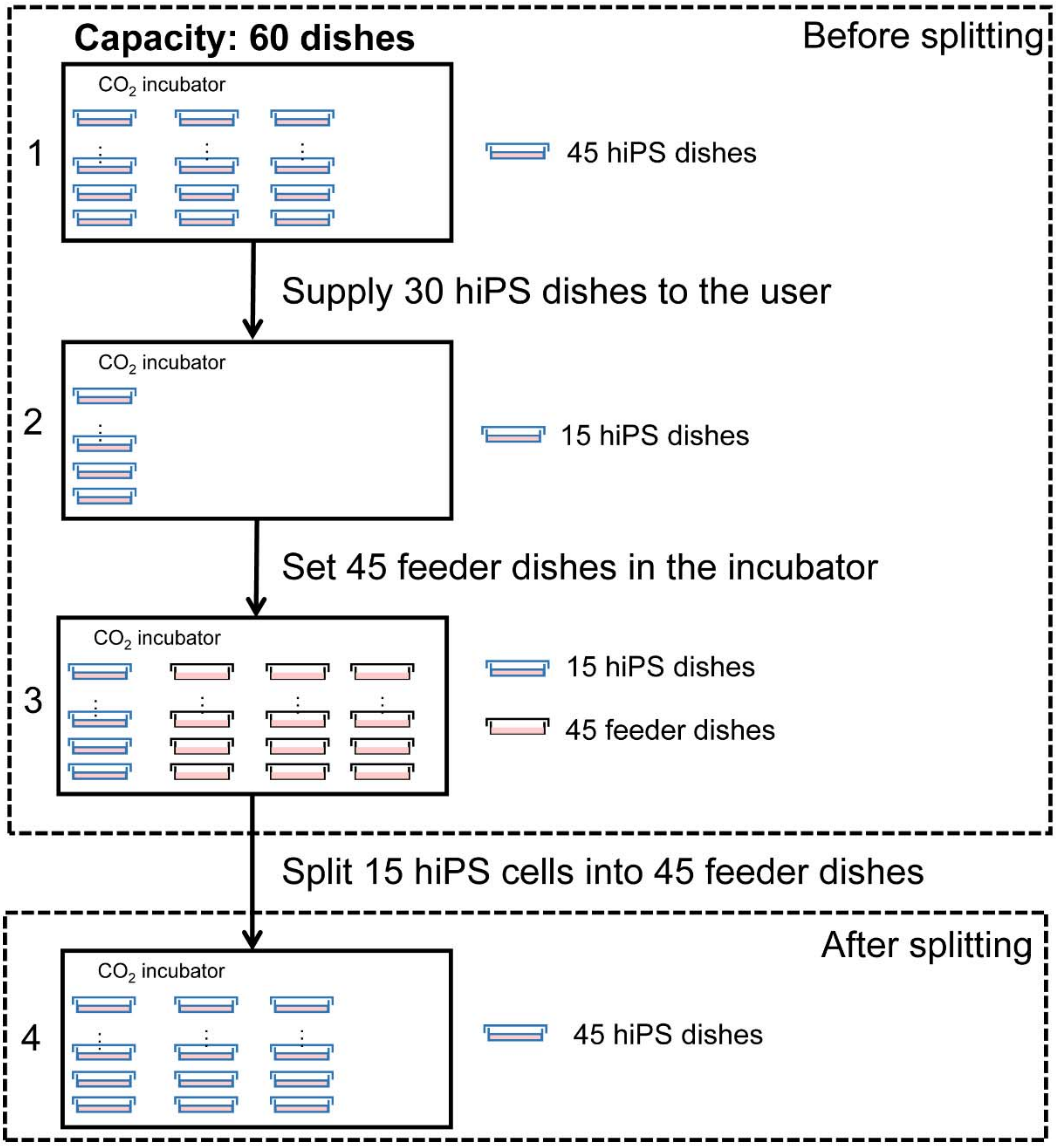

Supplementary Figure 1: Schematic illustration of the splitting procedure. 1: forty-five hiPS dishes were cultured in the incubator. 2: thirty dishes with hiPS cells were supplied to the users. 3: Before the splitting, forty-five feeder dishes were set in the incubator. Sixty dishes were in the incubator. 4: HiPS cells in the remaining fifteen dishes were split into forty-five feeder dishes.

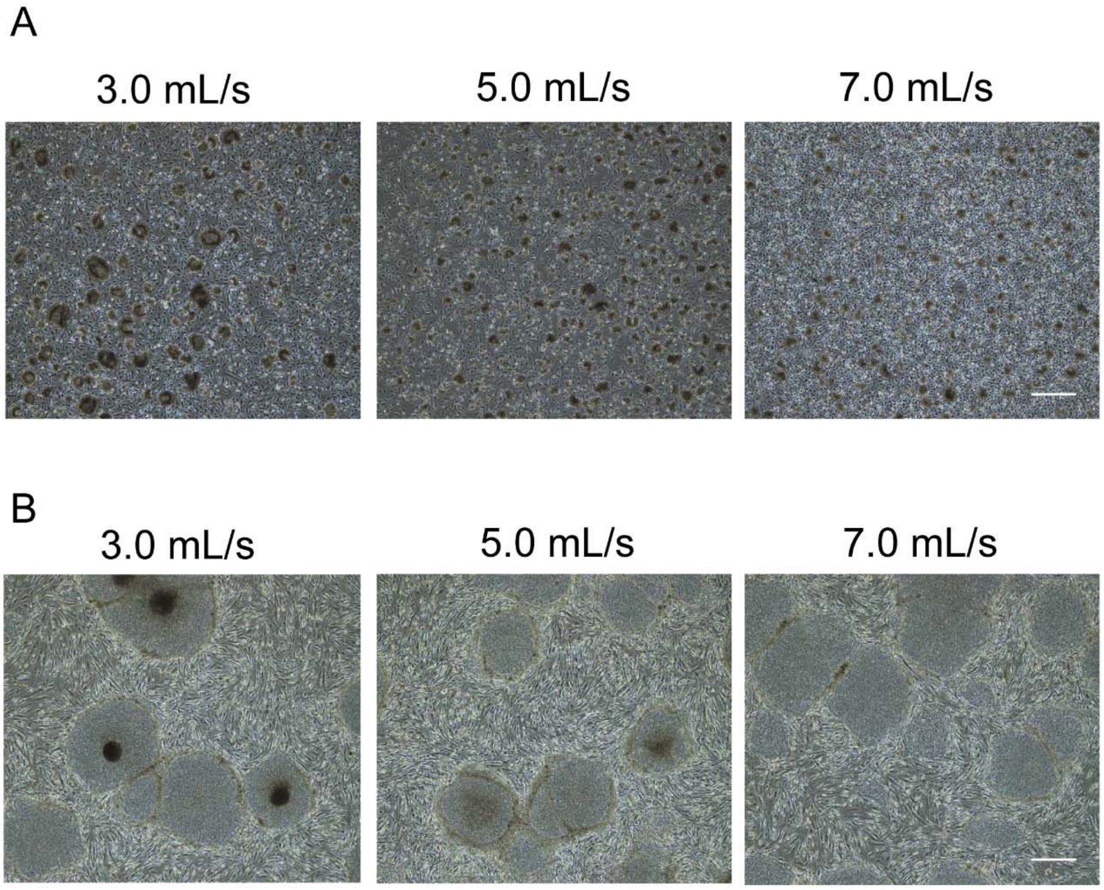

Supplementary Figure 2: Optimization of the pipetting velocity. Clumps' size of hiPS cells were controlled by flow rates of medium in the pipetting procedure. The pipetting velocity was set to 3.0 mL/s, 5.0 mL/s, or 7.0 mL/s. A, B: Phase micrographs of hiPS cell clumps (colonies) immediately after dividing (A) and after three days (B). Scale bar: 500 μm.

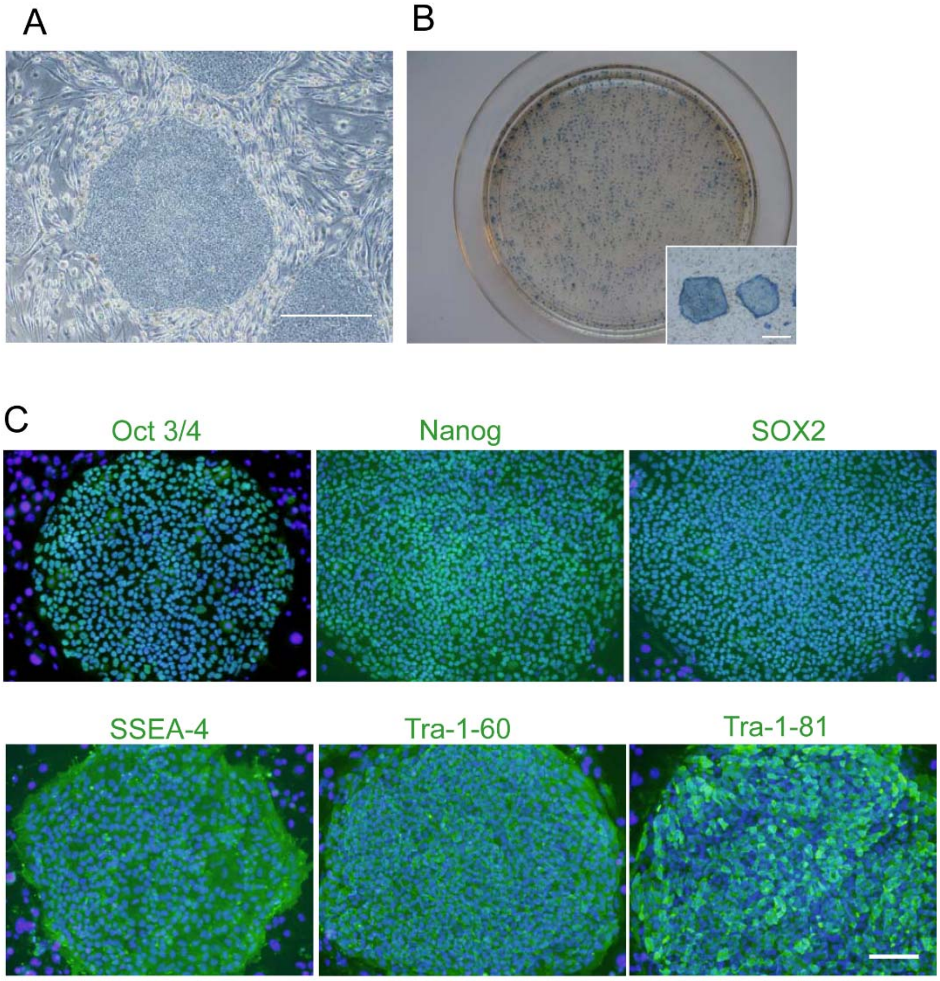

Supplementary Figure 3: Passaging of hiPS cells (253G1) using Dispase. HiPS cells were automatically subcultured using 2U/mL Dispase (Roche Applied Science, Mannheim, Germany). A: Phase contrast micrographs of hiPS cells on day 3. B: Alkaline phosphatase staining of hiPS cells on day 3. C: Fluorescent micrograph of hiPS cells on day 3. Cells were immunologically stained with antibodies against OCT 3/4, NANOG, SOX2, SSEA-4, TRA-1-60, and TRA-1-81. Cell nuclei were stained with Hoechst 33258. Scale bar: 500 µm (A, B), 200 µm (C).

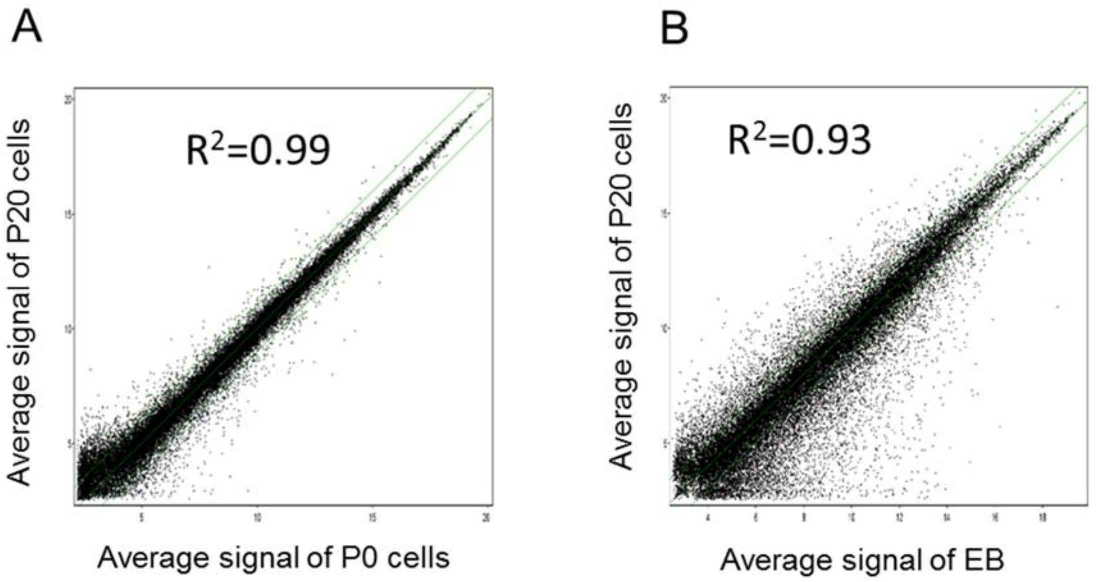

Supporting Figure 4: Scatter plots of averaged global gene expression (n =3). Averaged signal of hiPS cells passaged 20 times (P20 cells) was compared with those of unpassaged cells (P0 cells) and differentiated cells (EB).

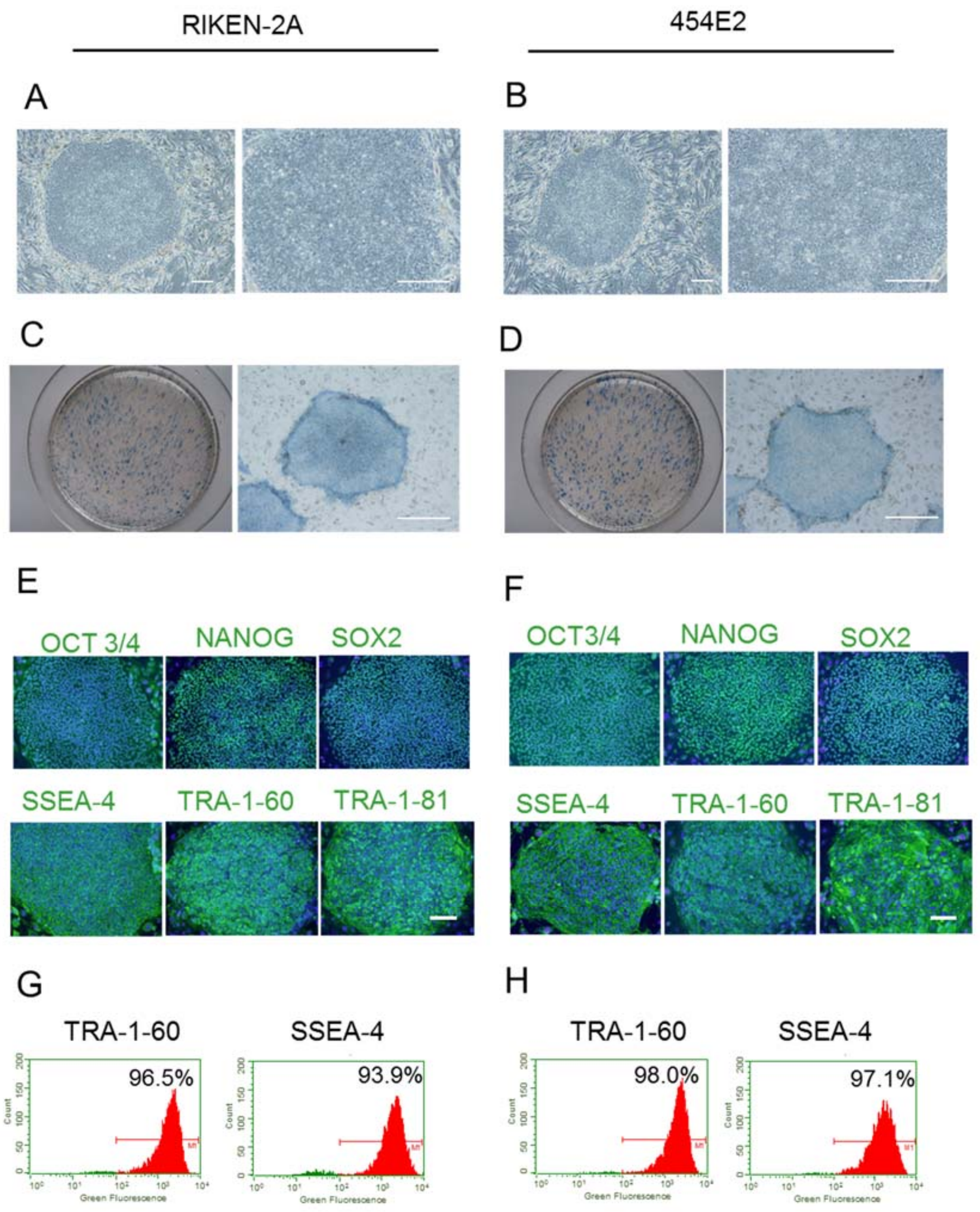

Supplementary Figure 5: Automated maintenance of other hiPS lines (RIKEN-2A and 454E2). A, B: Phase micrographs of hiPS cells (RIKEN-2A (A) and 454E2 (B)) on day 3. Scale bar: 200 µm. C, D: Alkaline phosphatase staining of hiPS cells (RIKEN-2A (C) and 454E2 (D)) on day 3. Scale bar: 500 µm. E, F: Fluorescent micrograph of hiPS cells (RIKEN-2A (E) and 454E2 (F)) automatically passaged 4 times. Cells were immunologically stained with antibodies against OCT 3/4, NANOG, SOX2, SSEA-4, TRA-1-60, and TRA-1-81. Cell nuclei were stained with Hoechst 33258. Scale bar: 200 µm. G, H: Representative histograms of FACS analysis.

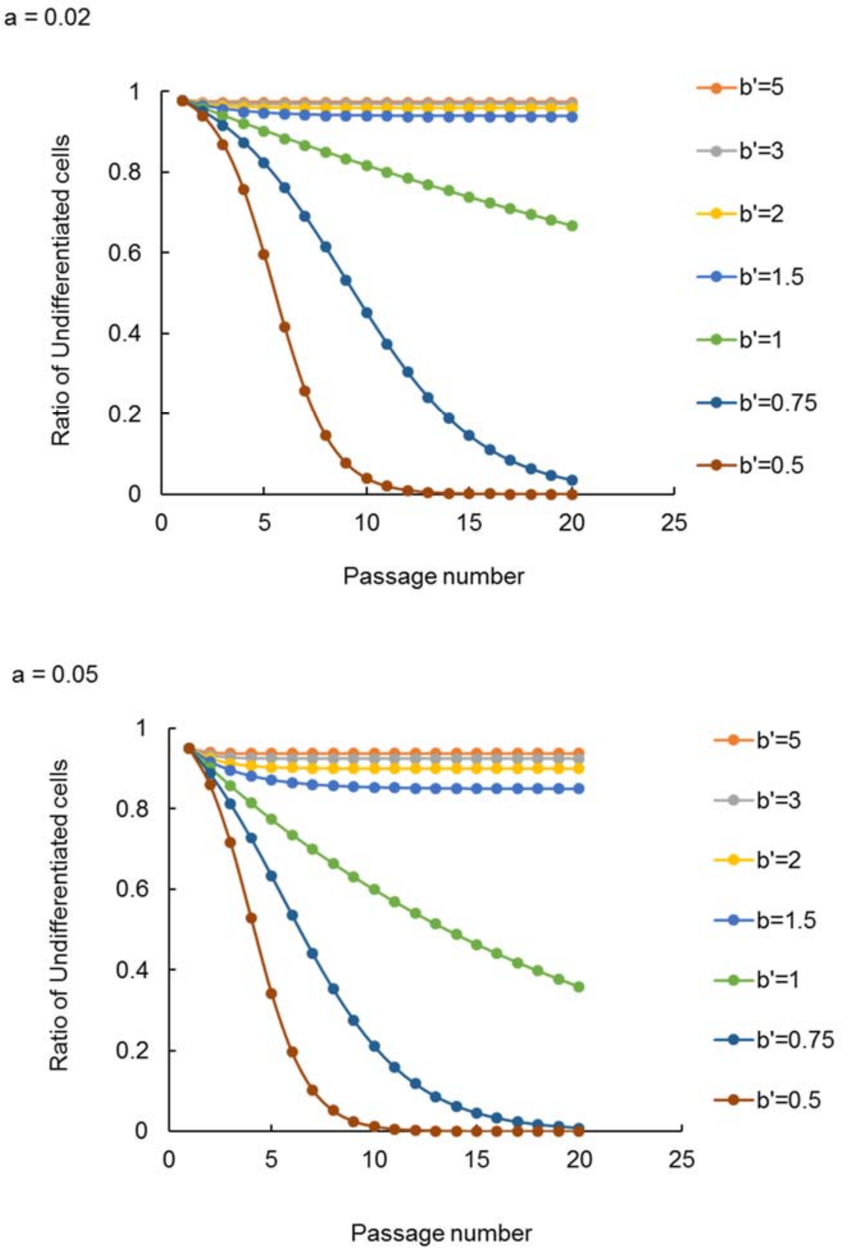

Supplementary Figure 6: Ratio of undifferentiated cells and total cells during a long-term culture. a: Probability of differentiation of undifferentiated cells during 3 days. b' : The ratio of growth rates of undifferentiated and differentiated cells. This figure is related to Supplementary discussions.
